# Decoding dynamic lipase trajectory patterns and in-hospital mortality in acute pancreatitis: insights from machine learning in intensive care units

**DOI:** 10.1186/s40001-025-03299-4

**Published:** 2025-10-22

**Authors:** Yingyi Li, Xiaoqiang Liu, Xiaodong Zhu, Chanchan Lin, Qilin Yang, Zicheng Huang, Yisen Huang

**Affiliations:** 1 Department of Gastroenterology, The First Hospital of Quanzhou Affiliated to Fujian Medical University, Quanzhou, China; 2 Department of Critical Care, The Second Affiliated Hospital of Guangzhou Medical University, Guangzhou, China

**Keywords:** Acute pancreatitis, Lipase trajectories, Mortality, Intensive care, Machine learning

## Abstract

**Background:**

Serum lipase levels are crucial biomarkers in acute pancreatitis (AP), yet their dynamic patterns and prognostic implications remain incompletely understood. This study aimed to identify distinct lipase trajectory phenotypes and evaluate their association with in-hospital mortality in AP patients.

**Methods:**

We conducted a retrospective analysis of 834 AP patients from the MIMIC-IV database using latent class trajectory modeling (LCTM) to identify distinct lipase trajectory phenotypes. Cox regression models, adjusted for demographics, comorbidities, clinical therapies, and critical illness markers, were employed to assess the association between trajectory classes and in-hospital mortality.

**Results:**

Three distinct lipase trajectory phenotypes were identified: Class 1 (*n* = 543) with consistently low levels, Class 2 (*n* = 51) with extremely high and variable levels, and Class 3 (*n* = 240) with moderately elevated levels. Class 2 patients were significantly older (66.8 ± 17.6 years) and had higher comorbidity burden (CCI: 5.6 ± 3.0). In-hospital mortality rates were 12.2%, 17.6%, and 19.2% for Classes 1, 2, and 3, respectively. After comprehensive adjustment, both Class 2 (HR: 2.21, 95% CI 1.04–4.71, *p* = 0.042) and Class 3 (HR: 1.61, 95% CI 1.08–2.40, *p* = 0.022) showed significantly higher mortality risk compared to Class 1.

**Conclusions:**

Dynamic lipase trajectory patterns in AP patients demonstrate distinct phenotypes with significant prognostic value for in-hospital mortality. These findings suggest that monitoring lipase trajectories may enhance risk stratification and guide clinical management in AP patients.

## Background

Acute pancreatitis (AP) is a common inflammatory disorder of the pancreas, characterized by abdominal pain, elevated serum lipase, and inflammation. The pathophysiology of AP involves the premature activation of digestive enzymes within the pancreas, leading to autodigestion and inflammation of pancreatic tissue. The annual incidence of AP ranges from 13 to 49 cases per 100,000 individuals, as indicated by epidemiological data [1, 2]. AP represents a significant healthcare challenge, being one of the leading causes of gastrointestinal-related hospitalizations with approximately 300,000 emergency department visits annually in the United States [3]. The severity of AP can be classified into three categories according to the revised Atlanta classification: mild, moderately severe, and severe [4]. Approximately 20–30% of patients develop severe acute pancreatitis(SAP), often associated with organ dysfunction requiring intensive care [5]. The mortality rate varies significantly based on disease severity, with severe cases having mortality rates as high as 30% [6]. Timely prognostic appraisal is critical for guiding proper clinical interventions. Despite advances in understanding the pathogenesis and management of AP, challenges remain in predicting disease severity and outcomes. Several scoring systems, such as the Ranson criteria [7], Balthazar CT grading [8], Acute Physiology and Chronic Health Evaluation II (APACHE-II) [7], and Bedside Index for Severity in AP (BISAP) [9], have been developed to aid clinicians in predicting the severity of AP. Despite this, many of these systems are too intricate and not feasible for everyday clinical practice. In addition, these systems commonly show delays and may have constrained predictive capability. Hence, further investigation into novel prognostic tools is imperative to enhance the diagnosis, management, and outcome of AP.

Recent advances in AP management have led to a more nuanced understanding of the role of biomarkers in predicting disease severity and outcomes. Serum lipase is traditionally recognized for its diagnostic utility but not predictive value. Recently, serum lipase is traditionally recognized for its diagnostic utility in AP due to its superior sensitivity (85–100%) and specificity (95–100%) compared to amylase, which has lower specificity (70–90%) and shorter half-life. Recent studies further support lipase’s prognostic value: elevated lipase trajectories correlate with complications, such as pancreatic necrosis, organ failure, and mortality in diverse clinical contexts, including sepsis, COVID-19, and renal failure [10–12]. For instance, Kiyak et al. demonstrated that lipase elevation independently predicted ICU transfer and mortality in COVID-19 patients [11], while Bierma et al. identified lipase as a key predictor of severe pediatric AP [12]. In contrast, amylase lacks comparable prognostic evidence. Thus, lipase’s diagnostic robustness and emerging prognostic utility make it ideal for dynamic trajectory analysis.

Most previous studies have focused on single-timepoint measurements, while the dynamic changes of lipase levels over time may provide more prognostic information. We hypothesize that distinct lipase kinetic phenotypes reflect underlying pathobiological processes (e.g., ongoing necrosis vs. compartment syndrome) with differential outcomes. However, the dynamic patterns of lipase levels throughout the course of hospitalization and their relationship with clinical outcomes remain incompletely understood. The emergence of machine learning (ML) in healthcare has opened new possibilities for analyzing complex medical data and identifying patterns that may not be apparent through traditional statistical methods [13]. The integration of ML techniques with clinical data, particularly in intensive care settings, offers a unique opportunity to analyze the relationship between dynamic lipase patterns and patient outcomes. Previous studies have demonstrated the value of ML in analyzing complex medical data streams in critical care, but its application to lipase dynamics in AP remains unexplored [14].

Therefore, this study aims to analyze the dynamic patterns of serum lipase levels in patients with AP admitted to ICUs and their relationship with in-hospital mortality using ML approaches. By leveraging advanced analytical techniques, we seek to identify previously unrecognized patterns that might have prognostic significance and could influence clinical decision-making. This research could potentially bridge the gap between routine biochemical monitoring and clinical outcomes, leading to more informed and personalized management strategies for AP patients in critical care settings.

## Materials and methods

### Data source

This study conducted a retrospective cohort analysis using data from the extensive critical database, the Medical Information Mart for Intensive Care IV (MIMIC-IV). MIMIC-IV 3.0 is a unified longitudinal database from a single center, comprising information on 364,627 patients admitted to Beth Israel Deaconess Medical Center, with 94,458 ICU stays, covering the years 2008 to 2022 (https://mimic.mit.edu) [15]. The institutional review boards of both the Massachusetts Institute of Technology and BIDMC approved this study. Yingyi Li is responsible for data extraction and has been granted access to the MIMIC database (Record ID: 62389476).

### Study population and outcomes

**Fig. 1:**
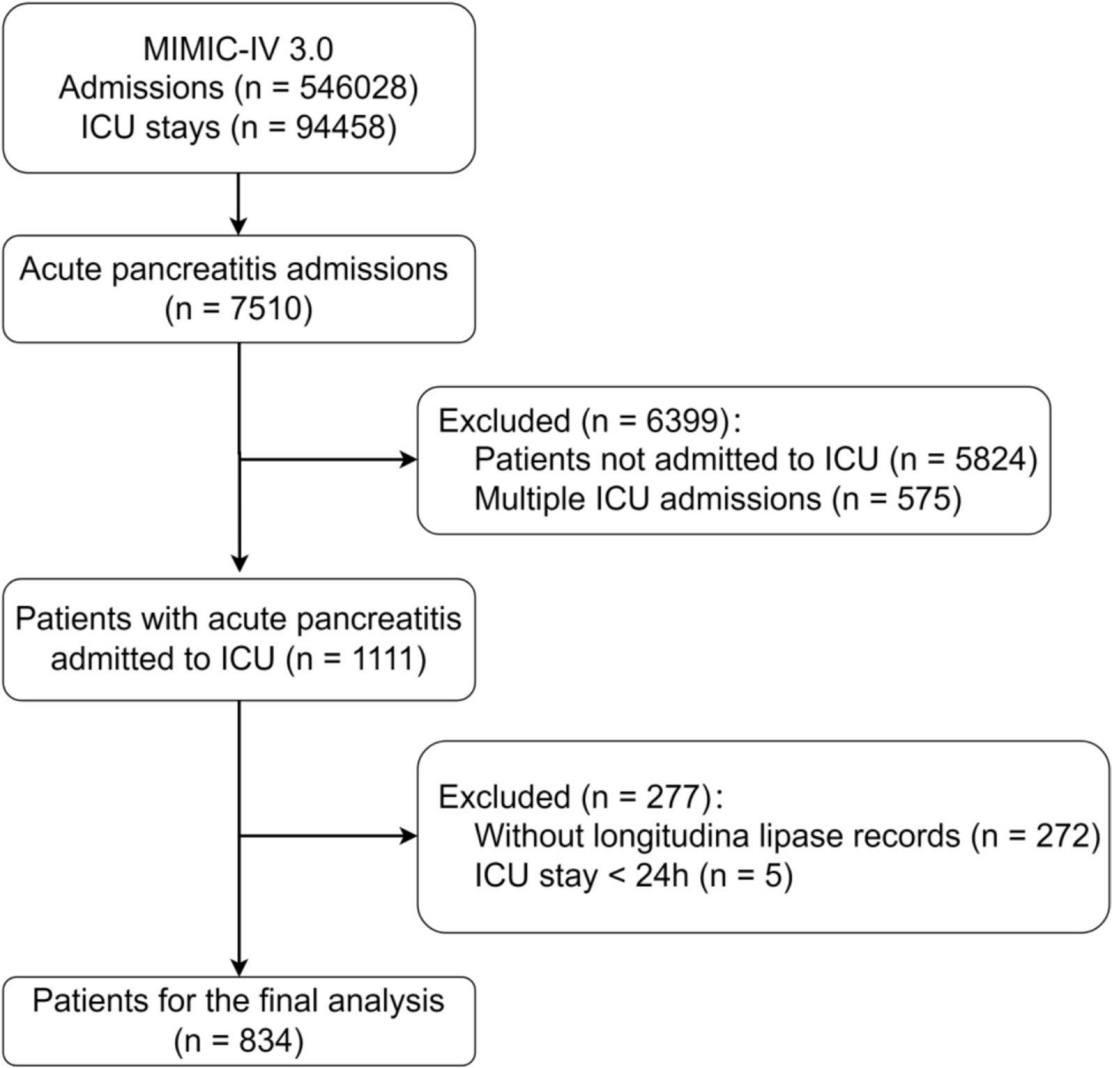
Diagram of patient selection for the study population

The inclusion criteria were: (1) patients diagnosed with AP according to the International Classification of Diseases, 9th Edition (ICD-9) code, (2) initial admission to the intensive care unit, and (3) age 18 years or older. Exclusion criteria for patients included: (1) absence of multiple lipase records, (2) missing data on in-hospital mortality, and (3) hospital stays of less than 24 h. A total of 834 ICU patients diagnosed with AP were extracted from the MIMIC-IV 3.0 database (Fig. 1). The primary outcome was in-hospital mortality.

### Data extraction

Data extraction from the MIMIC-IV database was conducted using Structured Query Language (SQL) in conjunction with the PostgreSQL tool. Demographic data, vital signs, complications, laboratory indicators, clinical evaluations, and treatment management were among the extracted information. Demographic data comprised age, gender, and ethnicity. The critical parameters consisted of respiratory rate, oxygen saturation (Spo2), temperature, blood pressure, and heart rate. Complications involved hypertension, diabetes, chronic pulmonary disease, myocardial infarction, congestive heart failure, renal disease, cerebrovascular disease, malignant cancer, liver disease, and peptic ulcer disease. The calculation of the Charlson Comorbidity Index (CCI) involves assigning points to 19 distinct comorbidities based on their relationship with 1-year mortality [16]. Other clinical assessments included the APACHE II [7] and Simplified Acute Physiology Score II (SAPS II) [17]. Therapeutic interventions comprised endoscopic retrograde cholangiopancreatography (ERCP), peritoneal drainage, mechanical ventilation, vasopressor administration, and continuous renal replacement therapy (CRRT). Lipase levels were collected for each patient between day 0 and day 7 following hospital admission. The normal reference range for lipase in the MIMIC-IV database: 0–60 IU/L. Besides lipase, the laboratory also assessed white blood cells, hemoglobin, red cell distribution width (RDW), total bilirubin, albumin, creatinine, urea nitrogen, glucose, sodium, potassium, calcium, anion gap, and lactate.

### Statistical analysis

Continuous variables, which are normally distributed, are denoted as mean ± standard deviation (SD). Continuous variables that were non-normally distributed are presented using the median with interquartile range (IQR). Categorical variables were presented as frequencies accompanied by their corresponding percentages. To tackle missing data problems, multiple imputations were carried out utilizing chained equations with 50 imputations to ensure unbiased estimates of the correlation between lipase trajectories and the outcome. For comparisons between different groups, categorical and continuous variables were tested for statistical significance using chi-square tests, Fisher’s exact tests, or Wilcoxon rank-sum tests, as appropriate.

We utilized the Latent Class Trajectory Model (LCTM) to ascertain the trajectories of lipase levels longitudinally. This model is a finite mixture model crafted to identify latent classes of individuals exhibiting similar progression patterns over time. Four LCTMs were constructed with two to five trajectories. The optimal number of trajectories was determined through the Akaike information criterion (AIC), Bayesian information criterion (BIC), sample-size adjusted BIC (SABIC), and entropy. Lower AIC, BIC, and SBIC values, coupled with higher entropy, signify superior model fit and discriminative capability. To ensure clinical significance and statistical robustness, a minimum average posterior probability discrimination level of ≥ 0.70 and a subgroup size threshold of 5% were set.

The association between lipase trajectories and outcomes was assessed through binary Cox regression models, adjusted for key covariates to avoid collinearity. In-hospital mortality was evaluated using Kaplan–Meier (KM) survival curves based on lipase trajectories, followed by a log-rank test. Subgroup analyses were conducted based on sex, age, liver disease, and renal disease to validate the results. Interaction across subgroups was assessed using the likelihood ratio test.

Statistical analyses were carried out using R software (version 4.2.2, http://www.R project.org, The R Foundation) and Free Statistics software version 2.1.

## Results

### Identification of lipase trajectory phenotypes

**Fig. 2:**
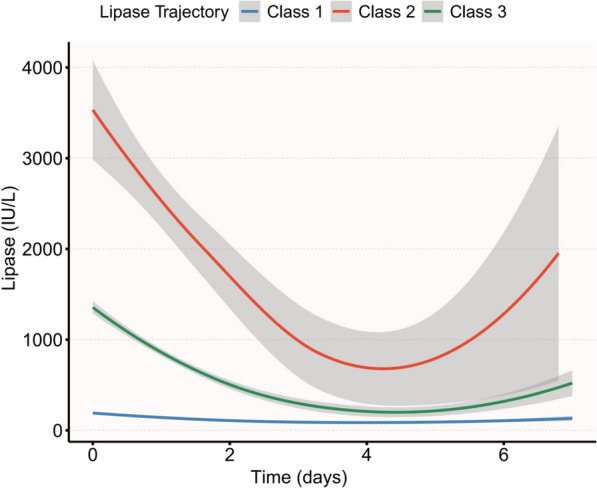
Trajectories and time distribution of lipase among acute pancreatitis phenotypes

In Table S1, the fit, discriminative power, posterior probabilities, and subgroup sizes of LCTMs with 2 to 5 trajectories are presented. Following a thorough analysis of this data, the LCTM with 3 trajectories was selected as the most suitable model. The analysis revealed three distinct subphenotypes: Class 1 (*n* = 543) characterized by consistently low lipase levels, Class 2 (*n* = 51) displaying extremely high and highly variable lipase levels, and Class 3 (*n* = 240) with moderately elevated levels showing intermediate variability (Fig. 2; Table 1).

**Table 1:** Comparison of lipase features within the three lipase-trajectory phenotypes

Characteristics	Total (*n* = 834)	Class 1 (*n* = 543)	Class 2 (*n* = 51)	Class 3 (*n* = 240)	*p*
Maximum lipase (IU/L)	388.5 (118.0, 1022.5)	161.0 (71.0, 368.5)	3170.0 (2167.0, 4380.0)	1189.5 (861.2, 1660.2)	< 0.001
Minimum lipase (IU/L)	62.0 (27.2, 150.8)	47.0 (24.0, 100.0)	201.0 (96.0, 985.5)	125.0 (39.8, 388.5)	< 0.001
Mean lipase (IU/L)	197.0 (83.2, 496.5)	112.0 (51.0, 195.5)	1565.7 (1160.5, 2327.6)	568.6 (378.9, 790.5)	< 0.001
Median lipase (IU/L)	138.2 (60.0, 366.5)	94.0 (44.0, 164.2)	1330.0 (551.5, 2233.0)	475.0 (151.5, 733.2)	< 0.001
Standard Deviation, Median (IQR)	164.2 (39.9, 421.6)	52.2 (19.1, 142.1)	1168.3 (809.7, 2048.0)	441.6 (302.2, 685.9)	< 0.001
Coefficient of Variation, Mean ± SD	0.8 ± 0.5	0.6 ± 0.4	1.0 ± 0.5	1.0 ± 0.5	< 0.001

Data are presented as median (interquartile range [IQR]) or mean ± standard deviation (SD)

Dynamic analysis of serum lipase levels indicated significant differences among the three classes (all *p* < 0.001). Maximum lipase levels in Class 2 were notably elevated, with a median of 3170.0 IU/L (IQR: 2167.0, 4380.0), compared to Class 1 (median: 161.0 IU/L, IQR: 71.0, 368.5) and Class 3 (median: 1189.5 IU/L, IQR: 861.2, 1660.2). Mean and median lipase levels in Class 2 were substantially higher than in the other classes, with a mean of 1565.7 IU/L (IQR: 1160.5, 2327.6) vs. 112.0 IU/L (IQR: 51.0, 195.5) in Class 1 and 568.6 IU/L (IQR: 378.9, 790.5) in Class 3. Furthermore, variability measures such as standard deviation and coefficient of variation of lipase values were significantly increased in Class 2 and 3 compared to Class 1.

### Demographic and clinical characteristics

Tables 2 and S2 show the demographic and clinical characteristics of 834 patients. Analysis of patient demographics showed that those in Class 2 were older, with an average age of 66.8 ± 17.6 years, compared to 58.3 ± 16.7 years in Class 1 and 59.8 ± 19.1 years in Class 3 (*p* = 0.003). Differences in baseline comorbidities and chronic disease burden indices were subtly noted, with the CCI slightly higher in Class 2 (5.6 ± 3.0) than in Class 1 (4.5 ± 3.0) and Class 3 (4.4 ± 2.7) (*p* = 0.032), indicating a greater burden of comorbid illness in Class 2. Vital signs such as heart rate, blood pressure, respiratory rate, Spo2, and temperature did not significantly differ. However, disease severity indices exhibited notable variability, with APACHE II scores being lowest in Class 1 (56.4 ± 28.7) and highest in Class 3 (64.5 ± 32.9), along with similar trends observed in SAPS II scores (*p* = 0.002 and *p* = 0.001, respectively).

**Table 2:** Basic demographics and clinical characteristics within the three lipase-trajectory phenotypes in MIMIC

Characteristics	Class 1 (*n* = 543)	Class 2 (*n* = 51)	Class 3 (*n* = 240)	*p*
Demographics				
Male, n (%)	314 (57.8)	26 (51.0)	130 (54.2)	0.462
Age, years	58.3 ± 16.7	66.8 ± 17.6	59.8 ± 19.1	0.003
Race (white), n (%)	343 (77.1)	31 (72.1)	153 (74.6)	0.328
Vital signs				
Heart rate (beats/min)	94.2 ± 17.7	91.9 ± 18.0	96.9 ± 19.6	0.083
SBP (mmHg)	122.2 ± 17.8	125.2 ± 20.5	122.2 ± 19.4	0.539
DBP (mmHg)	68.6 ± 13.3	66.1 ± 12.2	68.5 ± 13.9	0.435
MBP (mmHg)	82.8 ± 13.0	80.5 ± 12.2	82.7 ± 13.7	0.502
Respiratory rate (beats/min)	20.5 ± 4.4	21.5 ± 5.0	21.1 ± 4.5	0.082
Temperature (℃)	37.0 ± 0.6	36.8 ± 0.5	37.0 ± 0.7	0.133
Spo2 (%)	96.3 ± 2.1	96.1 ± 1.9	95.9 ± 2.2	0.050
Comorbidities, n(%)				
Hypertension	339 (62.4)	37 (72.5)	142 (59.2)	0.195
Diabetes	165 (30.4)	19 (37.3)	58 (24.2)	0.086
Myocardial infarct	58 (10.7)	6 (11.8)	23 (9.6)	0.853
Congestive heart failure	86 (15.8)	8 (15.7)	43 (17.9)	0.761
Cerebrovascular disease	31 (5.7)	2 (3.9)	16 (6.7)	0.784
Chronic pulmonary disease	108 (19.9)	6 (11.8)	44 (18.3)	0.352
Renal disease	82 (15.1)	13 (25.5)	41 (17.1)	0.147
Malignant cancer	42 (7.7)	6 (11.8)	10 (4.2)	0.059
Peptic ulcer disease	32 (5.9)	4 (7.8)	9 (3.8)	0.288
Liver disease	158 (29.1)	13 (25.5)	68 (28.3)	0.855
Charlson comorbidity index	4.5 ± 3.0	5.6 ± 3.0	4.4 ± 2.7	0.032
Disease severity				
APACHE II	56.4 ± 28.7	59.3 ± 29.1	64.5 ± 32.9	0.002
SAPS II	35.2 ± 15.7	38.9 ± 15.5	39.7 ± 17.8	0.001
Clinical treatments, n(%)				
Albumin use	247 (45.8)	16 (31.4)	118 (49.2)	0.068
ERCP	16 (2.9)	3 (5.9)	8 (3.3)	0.421
Peritoneal drainage	67 (12.3)	5 (9.8)	30 (12.5)	0.860
CRRT	56 (10.3)	7 (13.7)	46 (19.2)	0.003
Mechanical ventilation	249 (45.9)	15 (29.4)	114 (47.5)	0.057
Vasopressor use^a^	176 (32.4)	9 (17.6)	74 (30.8)	0.093
Laboratory indicators				
White blood cells (10^9^/L)	14.3 ± 8.3	13.9 ± 6.3	14.8 ± 8.0	0.625
Hemoglobin (g/dL)	11.0 ± 2.3	12.2 ± 2.4	11.8 ± 2.9	< 0.001
RDW (%)	15.1 ± 2.3	14.9 ± 2.3	15.0 ± 2.2	0.858
Total bilirubin (mg/dL)	0.9 (0.6, 2.8)	1.5 (0.8, 3.7)	1.2 (0.7, 3.2)	0.030
Albumin (g/dL)	2.8 ± 0.6	2.9 ± 0.6	2.9 ± 0.7	0.115
Creatinine (mg/dL)	0.9 (0.7, 1.6)	1.1 (0.9, 2.0)	1.1 (0.8, 2.2)	< 0.001
Urea nitrogen (mg/dL)	18.0 (11.0, 32.0)	22.0 (14.0, 36.0)	23.0 (14.0, 37.0)	0.003
Glucose (mmol/L)	150.6 ± 68.0	138.7 ± 70.1	139.6 ± 71.7	0.752
Sodium (mmol/L)	138.0 ± 5.4	137.3 ± 1.2	136.8 ± 6.6	0.653
Potassium (mmol/L)	4.0 ± 0.6	3.8 ± 0.4	4.4 ± 0.8	0.058
Calcium (mmol/L)	7.9 ± 1.3	7.8 ± 1.2	7.9 ± 0.8	0.972
Anion gap (mmol/L)	15.7 ± 5.3	17.9 ± 6.3	17.6 ± 5.9	< 0.001
Lactate (mmol/L)	1.7 (1.2, 2.6)	2.9 (1.9, 5.1)	2.2 (1.3, 3.6)	< 0.001
Clinical outcomes, n(%)				
In-hospital mortality	66 (12.2)	9 (17.6)	46 (19.2)	0.030

Data are presented as count (percent), median (interquartile range [IQR]) or mean ± Standard Deviation (SD)

*MIMIC-IV* Medical Information Mart for Intensive Care, *SBP* systolic blood pressure, *DBP* diastolic blood pressure, *MAP* mean arterial pressure, *SpO*_*2*_ pulse oximetry, *APACHE II* Acute Physiology and Chronic Health Evaluation II, *SAPS II* Simplified Acute Physiology Score II, *ERCP* endoscopic retrograde cholangiopancreatography, *CRRT* continuous renal replacement therapy, *RDW* red cell distribution width, *ICU* intensive care unit

^a^Vasopressors included norepinephrine, dopamine, epinephrine, phenylephrine, or vasopressin

### Association between lipase trajectory phenotypes and outcome

**Fig. 3:**
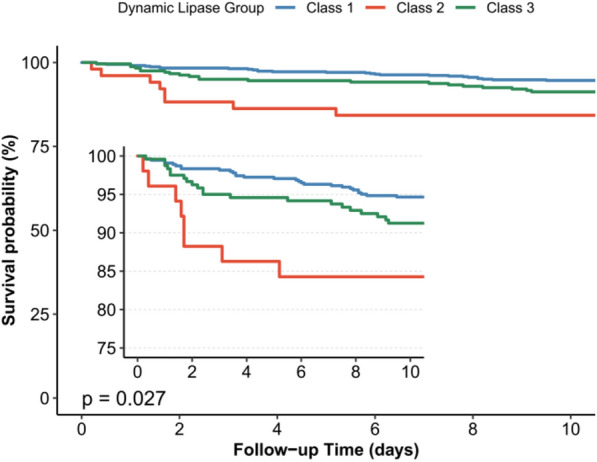
Kaplan–Meier survival curves of patients with three dynamic lipase trajectory phenotypes

The analysis of outcomes revealed significant differences in in-hospital mortality among the three lipase trajectory phenotypes (*p* = 0.030). Class 1 had a mortality rate of 12.2%, while Classes 2 and 3 had higher rates of 17.6% and 19.2%, respectively. The KM survival curve depicted notable prognostic variations among the distinct lipase trajectory subtypes (Fig. 3). Table 3 summarizes the associations between the three lipase trajectory classes and in-hospital mortality across five Cox regression models. In the crude analysis (Model 1), patients in Class 3 showed a hazard ratio (HR) of 1.63 (95% confidence interval [CI] 1.12–2.37, *p* = 0.012) compared to the reference group (Class 1). Conversely, Class 2, with an HR of 1.61 (0.80–3.23), did not reach statistical significance (*p* = 0.183). Following adjustments for demographic factors (Model 2), clinical comorbidities (Model 3), clinical therapies (Model 4), and markers of critical illness (Model 5), the association between Class 3 and in-hospital mortality remained stable and statistically significant (HRs ranged from 1.51 to 1.70 with *p* values between 0.008 and 0.041). Notably, in Model 4, Class 2 had an HR of 2.15 (95% CI 1.04–4.44; *p* = 0.042) and Class 3 had an HR of 1.51 (95% CI 1.02–2.23; *p* = 0.041), indicating a strong association with poor outcomes even after accounting for interventions and organ support measures. Model 5, which included additional laboratory markers, confirmed these associations with HRs of 2.21 (95% CI 1.04–4.71; *p* = 0.042) for Class 2 and 1.61 (95% CI 1.08–2.40; *p* = 0.022) for Class 3.

**Table 3:** Association between prognosis and lipase-trajectory phenotypes in cox regression models

Outcome	Class 1 (*n* = 543)	Class 2 (*n* = 51)		Class 3 (*n* = 240)	
		HR (95% CI)	*p*	HR (95% CI)	*p*
In-hospital mortality					
Model 1	1 (Ref.)	1.61 (0.80 ~ 3.23)	0.183	1.63 (1.12 ~ 2.37)	0.012
Model 2	1 (Ref.)	1.29 (0.64 ~ 2.61)	0.477	1.54 (1.05 ~ 2.25)	0.028
Model 3	1 (Ref.)	1.25 (0.62 ~ 2.54)	0.536	1.70 (1.15 ~ 2.49)	0.008
Model 4	1 (Ref.)	2.15 (1.04 ~ 4.44)	0.042	1.51 (1.02 ~ 2.23)	0.041
Model 5	1 (Ref.)	2.21 (1.04 ~ 4.71)	0.042	1.61 (1.08 ~ 2.40)	0.022

Model 1: Crude

Model 2: Adjusted for sex, age, race

Model 3: Renal disease, liver disease, congestive heart failure, malignant cancer

Model 4: Continuous renal replacement therapy, mechanical ventilation, vasopressor use, peritoneal drainage

Model 5: Simplified Acute Physiology Score II, white blood cells, red cell distribution width, total bilirubin, albumin, urea nitrogen, lactate

### Subgroup and sensitivity analysis

**Fig. 4:**
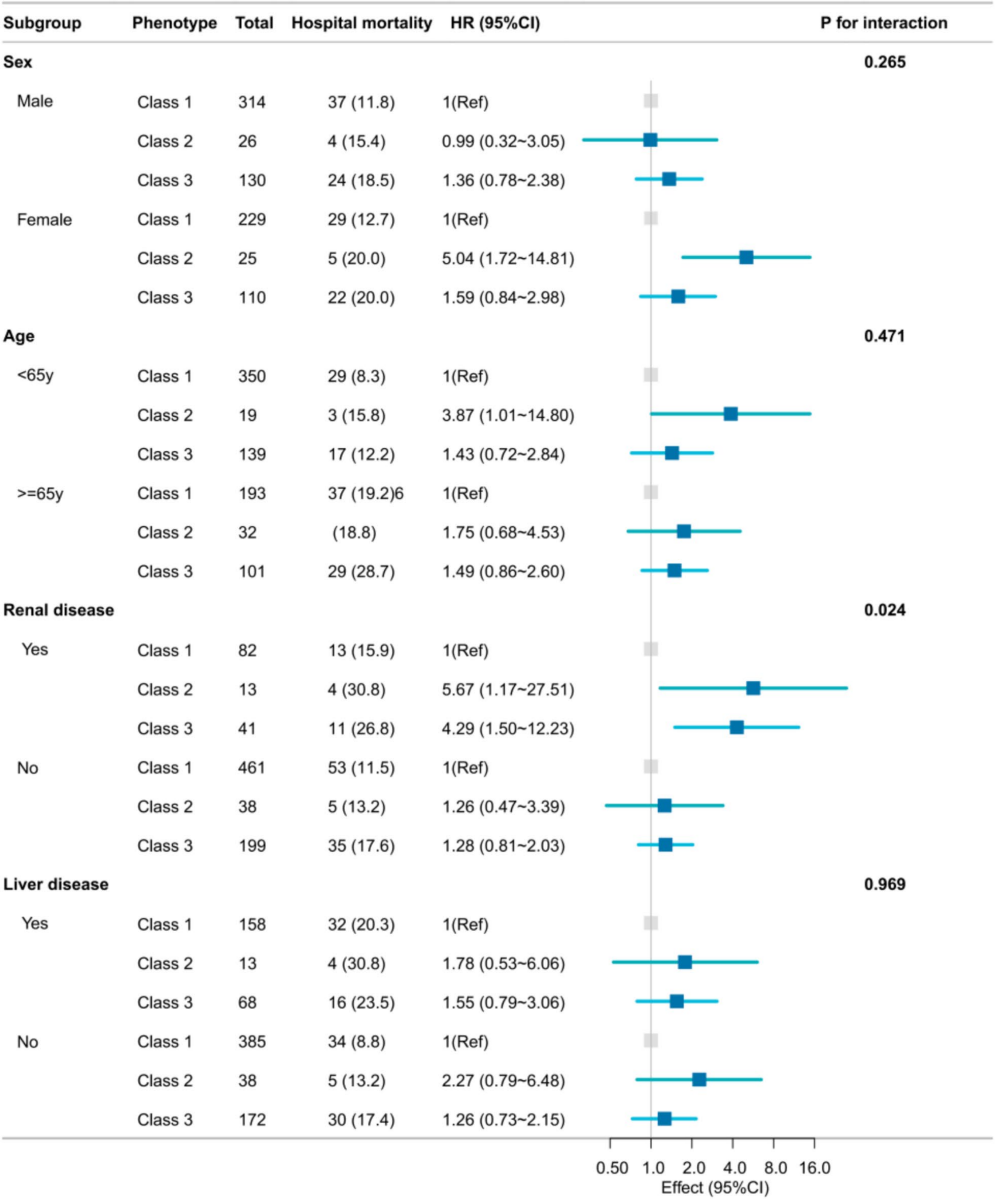
Forest plots of stratified associations between lipase trajectory phenotypes and in-hospital survival of patients with acute pancreatitis

In stratified analyses by sex, age, and the presence of liver and renal disease, the relationship between lipase trajectory phenotypes and in-hospital mortality risk remained stable across subgroups (Fig. 4). Despite statistically significant interactions (*p* < 0.05) between renal disease and lipase phenotypes, the clinical significance of these results may be diminished by the presence of multiple tests and the consistent directional patterns observed.

## Discussion

The study emphasizes the importance of dynamic biomarker trajectory assessment in AP, highlighting its advantages over traditional methods that depend on single or static serum lipase measurements. This dynamic approach is crucial for monitoring the disease's progression over time, especially in ICU settings. Our results indicate the presence of different lipase trajectory patterns in AP patients, with these patterns significantly linked to variations in disease severity and in-hospital mortality.

Lipase, primarily secreted by the pancreas, plays a vital role in fat breakdown into fatty acids and glycerol during digestion. Increased serum lipase levels in AP are a key marker of pancreatic inflammation and damage, emphasizing its diagnostic utility with higher sensitivity and specificity than amylase, often employed alongside lipase for a more thorough assessment. Lipase, primarily produced by the pancreas, plays a significant role in the digestive process by breaking down fats into fatty acids and glycerol. In the context of AP, elevated serum lipase levels are indicative of pancreatic inflammation and damage, making it a valuable diagnostic tool. The importance of serum lipase in diagnosing AP is underscored by its high sensitivity and specificity compared to other pancreatic enzymes, such as amylase, which is often used in conjunction with lipase for a more comprehensive assessment [18]. Emerging evidence indicates potential prognostic value in diverse clinical scenarios. Several studies have demonstrated the significance of lipase as a prognostic marker for AP in pediatric patients [12, 19, 20]. The predictive value of the lipase/lymphocyte ratio for mortality in patients with AP related to SARS-CoV-2 (COVID-19) was also observed in a previous study [21]. The role of lipase extends beyond pancreatitis. Hyperlipasemia is observed in several non-pancreatic digestive system illnesses, such as peptic ulcer, gastrointestinal bleeding, bowel necrosis or perforation, bowel obstruction, and inflammatory bowel disease [10]. Among patients in the ICU, hyperlipasemia is highly prevalent, with rates ranging from 40 to 57% [22–24]. Krisztina Eszter Feher et al. found that non-pancreatic hyperlipasemia is associated with a notable in-hospital mortality rate of 22.4% and the development of complications, such as sepsis and acute kidney injury [10]. Elevated lipase activity upon admission in COVID-19 patients, absent AP, as identified by Mevlut Kiyak et al., independently predicts a poor prognosis, indicating the need for ICU transfer, mechanical ventilation, and mortality [11]. The investigation of lipase as a prognostic biomarker extends to other domains like chronic renal failure. In Yingzhu's study, an association between serum lipase levels and disease progression was noted. Serum lipase serves as a valuable serological marker, reflecting clinical alterations in renal failure, with a tendency to rise as renal dysfunction advances [25].

Previous studies have mainly focused on single-timepoint lipase measurements, while this study highlights the prognostic value of dynamic lipase changes. Patients with AP may develop SAP even if they only have moderately elevated enzyme levels [26], indicating the potential limitations of relying solely on initial enzyme values for prognostic prediction. This phenomenon can be better captured through longitudinal monitoring. The application of latent class trajectory modeling—a machine learning approach—provided a nuanced understanding that goes beyond conventional static assessments. This method allowed for the identification of patient subgroups with distinct biochemical profiles that are not immediately evident from single-point measurements. Similar machine learning methodologies have been applied in recent research to predict in-hospital mortality in patients with acute pancreatitis-associated complications like acute kidney injury, with promising predictive accuracy [27]. This study, by applying latent class trajectory modeling to identify the lipase trajectory, revealed the following findings: Class 1, characterized by relatively low and stable lipase levels, exhibited the most favorable clinical outcomes with the lowest in-hospital mortality rate. In contrast, patients in Class 2, who demonstrated persistently high and variable lipase levels, experienced significantly higher mortality. Class 3, representing an intermediate pattern, also trended toward poorer outcomes compared to Class 1. From a pathophysiological perspective, the differences in lipase trajectories likely reflect heterogeneity in pancreatic injury, inflammatory cascade activation, and systemic organ involvement. Higher and more variable serum lipase values may indicate extensive pancreatic necrosis and a heightened systemic inflammatory response, both of which contribute to worse clinical outcomes.

The dynamic trajectory phenotypes identified in this study through unsupervised machine learning were significantly associated with in-hospital mortality. However, their translation into clinically practical tools requires further exploration. Nevertheless, we argue that the primary value of this study lies in providing unique insights that extend beyond discriminative power (AUC). While static scores offer a "snapshot" of a patient's status at a single timepoint, our trajectory phenotypes capture the "dynamic movie" of their clinical course. This dynamic information may offer added value in the following ways: (1) future electronic health record (EHR) systems could integrate algorithms for real-time trajectory analysis. If a patient's physiological parameters begin to deviate from a stable trajectory toward a known "deteriorating" phenotype, the system could trigger an early warning alert prior to changes in traditional scores, granting clinicians a crucial time window for intervention, (2) trajectory phenotypes could serve as a powerful complementary tool to existing scores for patient stratification, and (3) visual trajectory plots are an intuitive tool that could improve clinician–family communication. Physicians could use them to illustrate the clinical course and prognosis, facilitating shared decision-making.

Nonetheless, several limitations merit discussion. As a retrospective analysis based on data extracted from the MIMIC-IV database, the study is subject to inherent selection biases and potential confounding factors. The timing and frequency of lipase measurements were not standardized, and this variability may have influenced the trajectory modeling. Moreover, while the machine learning methods employed offer robust classification, external validation in prospective multicenter cohorts will be necessary to confirm the generalizability of these findings. Our findings are specific to critically ill AP patients requiring ICU care. Generalizability to milder cases warrants validation in non-ICU cohorts. In addition, this study has several limitations. First, the trajectory analysis is potentially subject to immortal time bias, because the outcome was in-hospital mortality. To be identified and assigned to a specific trajectory group, patients had to survive long enough to contribute multiple measurements. This means that patients who died very early after admission may have been under-represented or even excluded from the model. This represents an inherent methodological challenge, and future studies could employ more frequent data collection in the very early phase to validate and extend these findings. Moreover, future research should also consider integrating additional dynamic markers—such as inflammatory cytokines or hemodynamic parameters—to build more comprehensive predictive models.

## Conclusions

In summary, the application of machine learning techniques to dynamic serum lipase data has revealed that distinct trajectory patterns exist among patients with AP in the ICU. Our analysis demonstrates that patients with persistently elevated and highly variable lipase levels (Classes 2 and 3) exhibit greater disease severity and higher in-hospital mortality compared to those with lower and more stable levels (Class 1). These findings suggest that the incorporation of dynamic biomarker trajectory analysis into traditional risk stratification models could enhance clinical decision-making and ultimately improve patient outcomes. Further prospective validation and integration of additional clinical variables are warranted to optimize this promising approach.

## Data Availability

The data are available in the MIMIC repository, https://mimic.physionet.org/

## Abbreviations

AP: Acute pancreatitis
LCTM: Latent class trajectory modeling
MIMIC-IV: Medical Information Mart for Intensive Care IV
ICU: Intensive care unit
AIC: Akaike information criterion
BIC: Bayesian information criterion
SABIC: Sample-size adjusted Bayesian information criterion
HR: Hazard ratio
CI: Confidence interval
SAP: Severe acute pancreatitis
APACHE-II: Acute Physiology and Chronic Health Evaluation II
BISAP: Bedside Index for Severity in Acute Pancreatitis
ERCP: Endoscopic retrograde cholangiopancreatography
CRRT: Continuous renal replacement therapy
CCI: Charlson Comorbidity Index
SAPS II: Simplified Acute Physiology Score II
RDW: Red cell distribution width
SD: Standard deviation
IQR: Interquartile range
